# Radiocarbon dating the Greek Protogeometric and Geometric periods: The evidence of Sindos

**DOI:** 10.1371/journal.pone.0232906

**Published:** 2020-05-27

**Authors:** Stefanos Gimatzidis, Bernhard Weninger

**Affiliations:** 1 Austrian Academy of Sciences, Austrian Archaeological Institute, Vienna, Austria; 2 Institute of Prehistory, University Cologne, Köln, Germany; University of Oxford, UNITED KINGDOM

## Abstract

Mediterranean Early Iron Age chronology was mainly constructed by means of Greek Protogeometric and Geometric ceramic wares, which are widely used for chronological correlations with the Aegean. However, Greek Early Iron Age chronology that is exclusively based on historical evidence in the eastern Mediterranean as well as in the contexts of Greek colonisation in Sicily has not yet been tested by extended series of radiocarbon dates from well-dated stratified contexts in the Aegean. Due to the high chronological resolution that is only achievable by (metric-scale) stratigraphic ^14^C-age-depth modelling, the analysis of 21 ^14^C-AMS dates on stratified animal bones from Sindos (northern Greece) shows results that immediately challenge the conventional Greek chronology. Based on pottery-style comparisons with other sites, the new dates for Sindos not only indicate a generally higher Aegean Early Iron Age chronology, but also imply the need for a revised understanding of the Greek periodisation system that will foreseeably have a major impact on our understanding of Greek and Mediterranean history.

## Introduction

**Fig 1:**
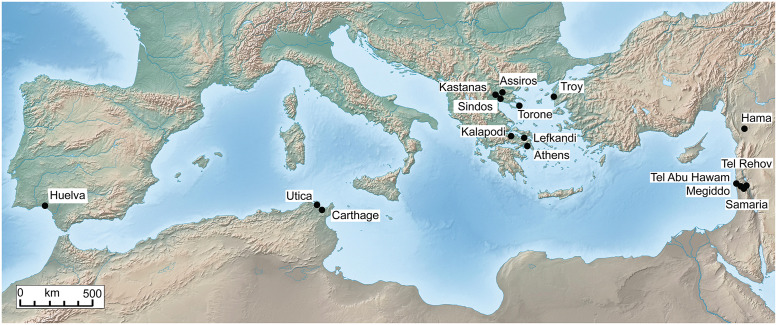
Map of the Mediterranean with sites mentioned in the text. Based on free vector and raster map data from @naturalearthdata.com. Constructed with Globalmapper^®^ Version 11 using Lambert Conformal Conic Projection.

In contrast to the Near East, where ancient cities often have the form of tell mounds, even the best excavated settlements in central and southern Greece have rarely yielded the long and continuous vertical stratigraphies that in other regions so readily support typo-chronological studies of their material inventories, at high temporal resolution. In Greece, the continuous settlement stratigraphies with well-dated successive layers, that cover many hundreds of years, are a privilege of the ‘northern periphery’ of the Aegean. In this region, dense networks of tell-based settlements developed continuously during the Bronze and Early Iron Age (Fig 1).

**Fig 2:**
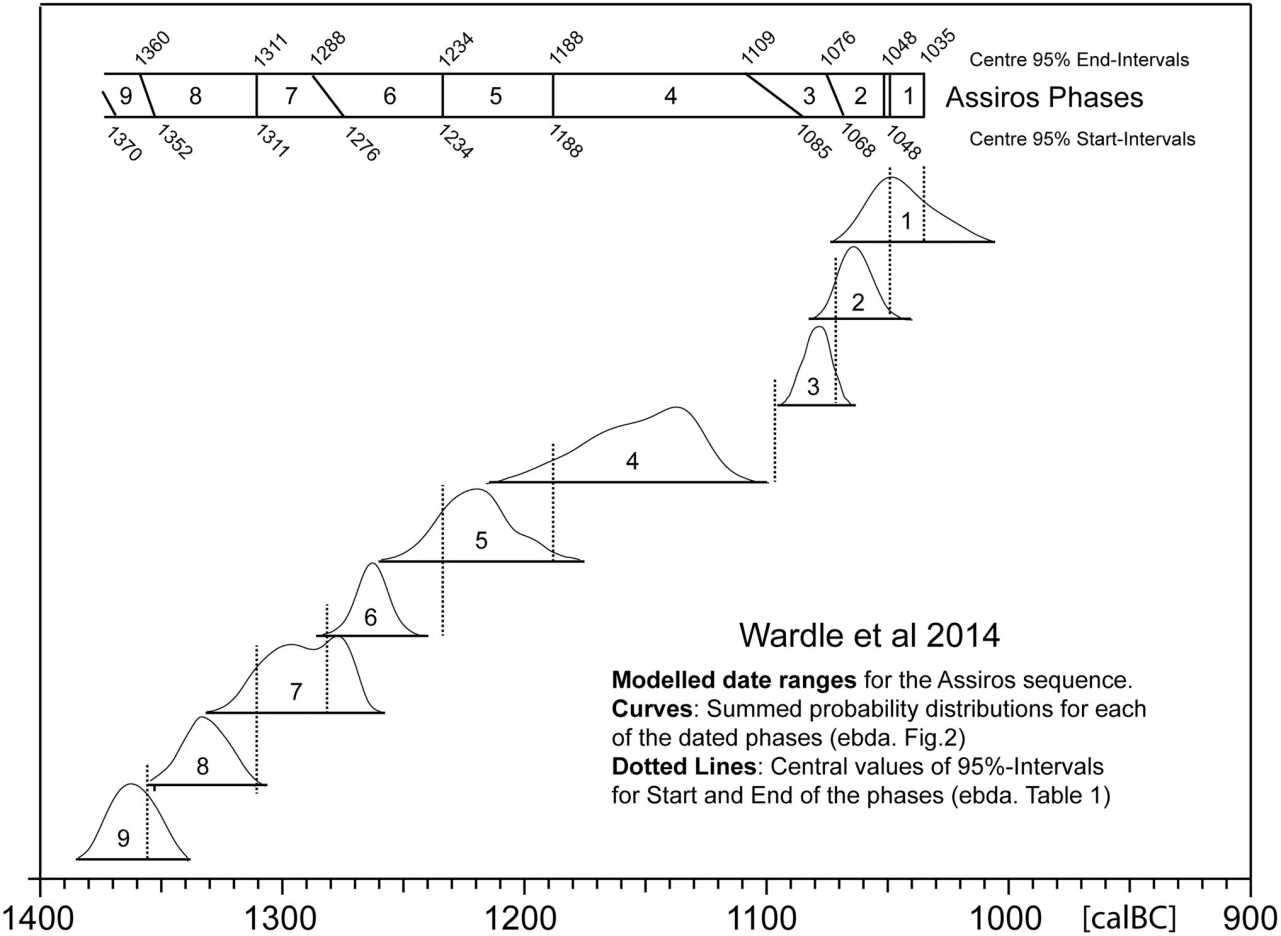
Bayesian age model for Assiros, redrawn from Wardle et al. 2014, Fig 2 including numeric ages taken from Wardle et al. 2014, Table 1. The modelled probability distributions for Phase 4 and 3 indicate a major hiatus in the stratigraphic sequence, which in reality does not exist.

In spite of the steadily increasing number of excavations, in central and southern Greece the Early Iron Age is still better known through cemeteries than settlements. This unfortunate deficiency in Greek archaeology has partly to do with the fact that the architecture of Early Iron Age settlements is indeed often badly preserved, e.g. at Athens and Corinth. Another relevant explanation is that the Early Iron Age settlements have attracted far less archaeological interest, if only because they seldom yield such impressive finds as their contemporaneous necropolises. In consequence, not only is there a general lack of interest in applying radiocarbon dating to the Aegean Early Iron Age, but even the well-excavated settlement sites–suffering as they do from short stratigraphic sequences–are lacking in the main archaeological requirement for high-resolution ^14^C-age modelling, which is the availability of an extended sequence of pottery data that would support either quantitative age-depth or pottery-based seriation of the ^14^C-measurements. In this respect, and what is sometimes overlooked, even highest-precision single ^14^C-measurements on short-lived samples (e.g. from well-defined burial or other contexts) cannot by themselves provide the envisioned high-resolution archaeological chronology. This would require advanced processing of multi-dimensional statistical data (i.e. interdisciplinary research), and ultimately the combination with quantitative pottery data, at best on some kind of metric-scale. Such metric-scaling (alias ‘*quantitative sequencing’*) is possible, trivially, by direct counting of tree-ring growth-sequences, but also for ^14^C-ages that are sequenced according to the time-factor derived from Correspondence Analysis (see below). A particular use of metric ^14^C-sequencing is by probabilistic (Monte Carlo) analysis of stratified ^14^C-ages from tell stratigraphies, as applied in the present paper [1] [2] [3]. In contrast, when based on ordinal-scaled (‘older-younger’) archaeological ^14^C-sequencing, as is the case for the majority of published Bayesian applications in archaeological research, the achieved chronology immediately runs danger of age-distortion due to the uncorrected convolution properties of the ^14^C-age calibration curve. As can also be derived from theoretical considerations, the more precise the archaeological ^14^C-ages are measured, the stronger their associated artificial age-distortions are likely to become, with actual values strongly depending on the contents of the archaeological sequence in relation to the shape of the calibration curve. A first confirmation for this forecasting is shown in Fig 2, where the application of Bayesian sequencing to a series of highly-precise ^14^C-ages from Assiros [4] that was measured on bone (N = 27) and combined with the ^14^C-ages on two tree-ring sequences has apparently produced an entirely artificial gap of at least 50 yrs length between phases 3 and 4. Presumably, this specific distortion is due to the inhomogeneity of the dataset, hence–ultimately–to the choice of an invalid Bayesian prior. Whatever its cause, the existence of this distortion due to inappropriate age-modelling invalidates the proposed radiometric updating of the Protogeometric vase from Assiros, which is of the very same magnitude. At the other methodological extreme, however, the solution cannot be the (assumed) model-neutral use of calibrated single ^14^C-ages, since in this case all that is achieved is repetitive stacking of the one-and-the-same calendric-scale interval, over and over again, with no achieved enhancement of the dating precision. In mathematical terms, the problem at stake is the non-commutativity of the underlying algebra of ^14^C-calibration, which complicates the analysis, in both cases, by effectively eliminating the otherwise so advantageous possibility of data-averaging with error-reduction [5]. The complications of (ordinal-scale) Bayesian age-modelling can be avoided, at large, by application of (metric-scale) stratigraphic age-depth modelling. This will be demonstrated below for the Sindos data.

In Early Iron Age research, if we now switch to the historical perspective, there are further reasons for the general reluctance towards using dating methods such as Radiocarbon or Correspondence Analysis. The main reason is the continuing confidence placed in the absolute dates that were gained by means of textual (historiographic) evidence. In historical terms, this confidence was the outcome of an early enthusiasm and a strong belief in the historicity of the written evidence, and which was eventually (and mainly unintentionally) transformed to become the supposedly scientific base of the now modern Greek chronology. This altogether quite disciplinary tradition in Aegean archaeology contrasts strongly with the highly intense debates on chronology in the Near East. During the last two to three decades, Near Eastern archaeologists, in particular in Israel, have undertaken a wide range of radiocarbon studies, which in many cases have led to the partial or even complete rejection of the authority of the textual evidence. This approach challenges the direct correlation of archaeological and historical data [6] [7] [8] [9], an approach yet to be customarily pursued in Aegean archaeology.

## The historical chronology of the Greek Early Iron Age

The scientific foundation of the conventional Greek Early Iron Age chronology has been, and still is, a much discussed and disputed issue. The beginning of the Protogeometric period, and the tripartite chronological definition of this, as well as the following Geometric period, is based to some large part on highly disputable historical evidence from the eastern Mediterranean. The method to assign absolute age-values to the Greek relative chronological system, which was based on pottery assemblages mostly from tomb contexts, was initially simple: single pottery finds from sites such as Tel Abu Hawam, Megiddo, Samaria, Hama [10], and more recently Tel Rehov [11] [12], were ascribed an absolute date that was obtained by the historical dating of the destruction layers of the respective site they were found in.

While there is practically no evidence that would support the chronological definition of the tripartite Protogeometric period, the transition from the Late Protogeometric to the Early Geometric is unceasingly based on a couple of sherds from a layer at Tel Abu Hawam, which has been variably dated. Similarly, a mere handful of sherds from Megiddo and Samaria has been used to describe, and date, the transitions from Early to Middle Geometric I (850 BC), Middle Geometric I to II (800 BC), Middle Geometric II to Late Geometric (760/750 BC). The same method, already partly used in construction of the Greek periodization by German scholars having worked in the necropolis of Kerameikos at Athens [13], was firmly established in Classical archaeology, following the comprehensive studies of Greek Protogeometric and Geometric pottery by Vincent R.d’A Desborough and Nicolas Coldstream [14] [15].

Aegean archaeology barely took into consideration the continuing discussions in Near Eastern archaeology about the historical dating of at least some of these sites that brought some large ambiguity about the validity of the evidence used in definition of the Greek Early Iron Age chronology. Although some of the eastern Mediterranean sites that yielded Greek pottery have recently also provided long series of precise ^14^C-determinations, such as Megiddo and Tel Rehov [16] [17] [18], these sites can still not be taken as safe anchor points for the Greek absolute chronology. This is typically due to the unclear contextual provenance of the randomly discovered Greek pottery finds [19].

Higher value is often placed on the chronological evidence that derives from the foundation dates of the Greek colonies in Sicily, which are still today widely used as anchor points for the absolute chronology of the Late Geometric and early Archaic period. This was due to an unwavering trust in the credibility of Thucydides, the most respected ancient Greek historiographer, who furnished both the foundation dates of some of the earliest Greek colonies, as well as the potential for correlation of the very same dates with the assumed earliest ‘colonial’ pottery found in the Greek establishments on Sicily [20] [21]. Single archaeological finds such as a scarab of the Egyptian king Bocchoris in a tomb at Pithekoussai may support the validity of this method, in certain cases [22] [23]. However, unexpectedly ‘earlier’ Greek pottery was soon found to have been used at some of those colonial sites, which was explained as the outcome of ‘precolonial’ contacts. Furthermore, there is still no consensus concerning the actual historicity and the sources of information available to Thucydides, who presents historical data concerning events that took place at least three centuries before his time [24]. Despite these open questions, the Sicilian evidence is usually considered as safer for the definition of the Late Geometric and Early Archaic periods than the available data from the eastern Mediterranean for the earlier Protogeometric and Geometric periods.

The construction of the relative and absolute chronology of the Greek Early Iron Age, which was the major outcome of Desborough’s and Coldstream’s studies, was not only based on the sequencing of Greek pottery by means of contextual approaches. Also integrated were the results of art historical methods according to the Zeitgeist. A good example, in this respect, is the main argument for the definition of the first part of the Late Geometric that predates the Greek colonisation. This was defined by Coldstream according to the artistic output of a specific Painter, the so-called Dipylon Master: Late Geometric Ia was thought to cover his early work and was therefore given a time span of ten years from 760 to 750 BC. The closely following Late Geometric Ib (750–735 BC) was associated with his late work, with the longer time span of 15 years given, if only to allow for his work together with other painters. While the earlier phases of the Greek Geometric period are based on ambiguous (but: at least) empirical ceramic evidence from the eastern Mediterranean, the beginning of Late Geometric and practically the entire Protogeometric periods are defined according to archaeological intuition [14] [15].

## Radiocarbon inconsistencies in the Greek Early Iron Age

Unfortunately, the radiocarbon evidence from the Aegean is neither consistent, nor itself sufficiently precise, to support the existence of proposed discrepancies between the historical and the ^14^C-based chronological systems. Although there are a number of ^14^C-dates from Submycenaean and Early to Middle Protogeometric contexts available at other sites apart from Assiros, e.g. at Kastanas [25] and Torone [26] in the northern Aegean, as well as at few other sites in the central and southern Aegean [27], if we take a closer look at the achieved dating precision (in statistical terms) or otherwise at the sampling and documentation methods (in archaeological terms), the quality of these ^14^C-series is insufficient to support further discussion.

## The radiocarbon dates of Sindos

**Fig 3:**
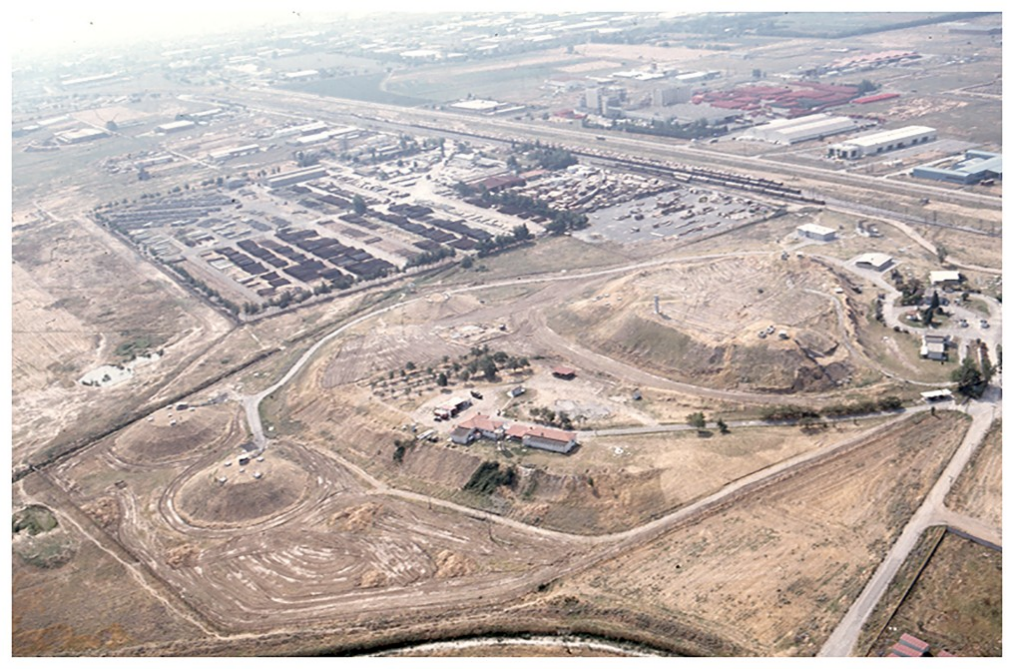
Aerial view of the tell-based settlement of Sindos, northern Greece comprising a higher and a lower artificial mound. The two conical structures to the left are modern (photo taken in 2001).

The tell mound of Sindos is located some 20 km west of Thessaloniki in northern Greece (40°42’01°N and 22°47’35°E) (Fig 3). This is one of the largest and most complex formed tell settlements in central Macedonia. A settlement system comprising almost exclusively tells developed in the river valleys and coastal plains of this region during the Bronze and Early Iron Age. These settlement sites have the form of conical mounds, known as toumbas in regional archaeology, during the Bronze Age. From the beginning of the Early Iron Age onwards new and larger settlement mounds emerged. They have the form of extended and elevated plateaus that are known as tables. These later tells emerged either independently as new settlement sites or adjacent to older toumbas as means to expand habitation surface.

Excavations conducted in the 1990’s and early 2000’s at several parts of the ancient settlement of Sindos revealed long stratigraphic sequences–particularly of the Early Iron Age. These excavations highlighted all major episodes in this tell’s formation: adjacent to an ancient core settlement that initially—during the Bronze Age—probably had the form of a conical tell (toumba), in the following Early Iron Age there emerged not one but two large tells, both with flat surfaces (tables) and both significantly expanding the settlement’s habitation area. Such complex processes in the formation of Macedonian tells, that typically involve extensive levelling and other major earthworks, mainly the construction of extensive terraces made of clay, are not only documented at Sindos, but are analytically presented in the final publication of the excavations, and hence available in all detail necessary for further research [28].

### Complexities in tell formation and depositional history

The formation of a tell mound is a complex depositional process that may hide pitfalls in age-depth modelling due to deep stratigraphic reworking. Sindos presents several characteristic cases of such complex processes in tell formation. For example, the deposition of Late Bronze Age layers immediately below the superficial Late Archaic layers at the top of the toumba may seem puzzling, if someone considers that long sequences of Early Iron Age levels were attested at almost all other parts of the settlement. Apparently debris and layers of this period were missing from the top of the toumba due to continuous levelling works. Furthermore, according to conventional pottery dates, following phase 4 at Sindos we might expect the existence of a major hiatus that dates from the end of the 8^th^ century BC. Further, since the layers of the immediately overlying phase 3 were deposited after the mid-6^th^ century BC, acceptance of the conventional dates would imply that a 150 yrs long hiatus marked the settlement history of Sindos during the Archaic period. Below, we will return to our many suspicions that there is *something wrong* with the conventional historical chronology, when applied to Sindos, and this is evident without application of radiocarbon dating. For the moment, let us begin by analysing the strengths and weaknesses of radiocarbon dating, quite specifically, in the context of a complex tell stratigraphy.

One main concern for application of radiocarbon dating at tell sites pertains to the possibility of stratigraphic reworking of the dated samples. In this respect what immediately comes to mind is the often encountered (upward) reworking of older materials to younger levels. Indeed, since this upward reworking runs parallel to the growth direction of the tell, this would appear the most ‘natural’ direction for the large majority of disturbances. Further, such upwards directional-modelling of tell deposits also corresponds to what may be called the main ‘axiom’ of stratigraphic analysis, that is: the deposits are best dated by their ‘youngest’ inventary. Equally possibly, however, is the stratigraphic re-working in the opposite direction i.e. from younger to older levels, and this would be the immediate (and equally ‘natural’) consequence of the often large-scale and systematic site-management activities of the tell-community. At the Neolithic site of Shir in Syria, for example, some ~45% of all (N = 40) ^14^C-dated grain samples from settlement pits have ages 100–200 yrs *younger than* the ^14^C-ages of (incorrectly assumed) ‘contemporaneous’ (i.e. same depth) settlement layers [29]. Actually, such an unexpected inversion of the more accustomed upwards direction of re-deposition may help to explain one of the major still-existing discrepancies between calibrated ^14^C-ages and historical dates. Namely, given the accumulation rate of ~1–2 cm/yr for a typical tell-mound, in combination with the depth of ~1 m for a typical storage pit, such secondary re-deposition of dated materials in younger-> older direction would provide a ‘natural’ explanation at least in quantitative terms for the observed offset of ~100 yrs between the Egyptian historical chronology and the calibrated ^14^C-ages from Tell el Daba [30]. In a nut-shell, resolving such issues is of importance, since enforcing a chronology at fault in one field of research has immediate consequences for other research fields.

Of course, we cannot exclude the occurrence of either sedimentational effects (upwards or downwards) also at Sindos, after having indeed identified a number of possible cases. Nevertheless, it is difficult to imagine that any such material re-deposition would explain all the discrepancies with the historical chronology that we observe at Sindos, and which would allow us to (artificially) maintain its validity vis-a-vis the emerging evidence of its faults (cf. below). Maintaining its validity has consequences, for example, it would imply *(ad hoc*) that the (historically dated) pottery sherds and the (^14^C-dated) animal bones–which show an excellent linear sequence (see below)–would have undergone some kind of systematic vertical separation, in the order of ~1 m, but for which there is no presently ascertained physical or cultural process. The point hereby is that, even if there did exist some unrecognised (say ~1 m deep) storage pits, and even if these pits were in-filled from above, this would not affect the original association of the (historically dated) pottery sherds with the (^14^C-dated) animal bones. The same argument would apply, similarly, even for the alternative case that the pottery and bones do not derive from some unrecognised deep-storage pit but from some other of the many types of tell-management deposits. We may expect the original association of pottery and bones to remain intact, under the large majority of depositional conditions, unless we assume the existence of some material-discriminating physical or cultural process that would act differently for sherds and broken bones (but which is unlikely, as noted above, if only with the exception that dogs seldom eat sherds). Another point-at-stake is that–in clear contrast to the aforementioned case of Shir–the ^14^C-data at Sindos do not show any unusually wide spread of ^14^C-ages, that would be indicative for the postulated inter-level (or inter-phase) sample movement.

Nonetheless, an important issue in age-modelling is to critically contrast the primary (sedimentological) site-formation processes with the (cultural) definition of settlement-formation according to archaeological phases/periods. Inevitably, the sedimentological sequence of layers will seldom find a match in the temporal sequence of phases/periods. At Kastanas, for example, a site in close vicinity to Sindos, some buildings were destroyed only a few years or decades after erection, whereas others (e.g. level 12) have a life-span of more than a century before being levelled for purposes of rebuilding [31]. In contrast to the applied linear age-depth modelling, at Sindos the actual tell-formation is indeed (possibly) not well-represented as series of continuous, successive, and equilength sedimentological episodes.

### Sampling methods and strategies

Despite all complexities in tell formation, archaeological exploration at such settlement sites is often accompanied with the welcome opportunity of working with successive destruction layers, that have clearly distinct ash and other burnt materials covering well preserved artefacts on surfaces of use. The house-floor assemblages that were found directly beneath thick layers of ashes and collapsed walls do not necessarily provide direct comparisons with the Pompeii-like systemic inventories that have both rapid and abrupt depositional qualities (Pompeii premise). They may nevertheless be taken as assemblages of artefacts, archaeobotanical and faunal material that were deposited and probably also used in the same period. At Sindos, such near-ideal stratigraphic conditions for radiocarbon sampling are given in a number of instances. An example is the fire-destruction of phase 7, from which six short-lived bone samples were sampled (cf. Table 1).

**Table 1:** Radiocarbon dates of sindos.

ID	Lab-Code	^14^C-Age [BP,1σ]	Material	Species	δ^13^C [PDB ‰]	*C*:*N*	C [%]	Collagen [%]	Katalog Nr	Trench,Unit	Depth [cm]	Sindos Phase	Relative Chronology	Calendric Age [calBC, 68%]
1	MAMS-27018	2767±23	bone	Pig, scapula	-17,7	*3*,*2*	32,9	2,2	Kat.Nr.1	A.0 #58	380–395	Phase 6	Late Geometric Ib	910 ± 40
2	MAMS-27019	2552±23	bone	Bos taurus, mandibula	-16,1	*3*,*2*	33,1	3,9	Kat.Nr.2	A.0 #58	380–395	Phase 6	Late Geometric Ib	710 ± 80
3	MAMS-27020	2655±23	bone	Goat, metacarpus	-20,5	*3*,*2*	37,1	5,7	Kat.Nr.3	A.0 #60	404–424	Phase 7	Late Geometric Ia	820 ± 20
4	MAMS-27037	2607±24	bone	Sheep, tibia	-20,9	*3*,*6*	36,5	8,1	Kat.Nr.20	A.0 #59	395–404	Phase 7	Late Geometric Ia	790 ± 20
5	MAMS-27021	2687±23	bone	Sheep/goat, metatarsus	-18,4	*3*,*2*	44,3	2,5	Kat.Nr.4	A.0 #60	404–424	Phase 7	Late Geometric Ia	840 ± 30
6	MAMS-27022	2691±23	bone	Bos taurus (?), scapula	-19,8	*3*,*2*	43,1	5,8	Kat.Nr.5	A.0 #60	404–424	Phase 7	Late Geometric Ia	840 ± 30
7	MAMS-27023	2704±25	bone	Bos taurus, radius	-21,6	*3*,*2*	36,4	4,8	Kat.Nr.6	A.0 #61	424–444	Phase 7	Late Geometric Ia	850 ± 30
8	MAMS-27024	2696±22	bone	Bos taurus, humerus	-19,1	*3*,*4*	36,5	4,6	Kat.Nr.7	A.0 #61	424–444	Phase 7	Late Geometric Ia	840 ± 30
9	MAMS-27025	2757±23	bone	Bos taurus (?), metatarsus	-19,8	*3*,*5*	31,9	4,2	Kat.Nr.8	A.0 #66	493–508	Phase 8	Middle Geometric II	890 ± 40
10	MAMS-27026	2761±22	bone	Sheep/goat, femur	-20,9	*3*,*4*	23,7	4,5	Kat.Nr.9	A.0 #66	493–508	Phase 8	Middle Geometric II	900 ± 40
11	MAMS-27038	2715±24	bone	Bos taurus, tibia	-15,2	*3*,*5*	31,5	5,6	Kat.Nr.21	A.0 #68	525–543	Phase 8	Middle Geometric II	860 ± 30
12	MAMS-27027	2812±23	bone	Pig, Humerus	-18,5	*3*,*4*	28,3	2,7	Kat.Nr.10	A.0 #83	573–584	Phase 9	Middle Geometric I	960 ± 30
13	MAMS-27028	2795±23	bone	Bos taurus, coxa	-15,2	*3*,*5*	31,5	5,6	Kat.Nr.11	A.0 #80	550–562	Phase 9	Middle Geometric I	950 ± 40
14	MAMS-27029	2777±24	bone	Sheep/goat, tibia	-20,7	*3*,*4*	29,8	2,8	Kat.Nr.12	A.0 #80	550–562	Phase 9	Middle Geometric I	930 ± 40
15	MAMS-27030	2837±23	bone	Bos taurus, metatarsus	-14,9	*3*,*5*	31,6	4,5	Kat.Nr.13	A.0 #90	612–623	Phase 10	Early Geometric	990 ± 40
16	MAMS-27031	2893±23	bone	Pig, tibia	-19,5	*3*,*5*	27,1	2,8	Kat.Nr.14	A.0 #90	612–623	Phase 10	Early Geometric	1070 ± 40
17	MAMS-27032	2779±23	bone	Pig, coxa	-20,6	*3*,*3*	31,8	5,3	Kat.Nr.15	A.0 #92	623–637	Phase 10	Early Geometric	930 ± 40
18	MAMS-27033	2877±23	bone	Pig, mandibula	-19,8	*3*,*5*	31,8	5,3	Kat.Nr.16	A.0 #92	623–637	Phase 10	Early Geometric	1050 ± 40
19	MAMS-27036	2847±24	bone	Pig, tibia	-20,5	*3*,*1*	25,7	4,2	Kat.Nr.19	A.0 #92	623–637	Phase 10	Early Geometric	1000 ± 40
20	MAMS-27034	2880±24	bone	Sheep/goat, tibia	-19,9	*3*,*5*	30,5	5,6	Kat.Nr.17	A.0 #105	673–685	Phase 11	Late Protogeometric	1060 ± 40
21	MAMS-27035	2809±23	bone	Bos Taurus, calcaneus	-12,1	*3*,*5*	30,3	4,8	Kat.Nr.18	A.0 #105	673–685	Phase 11	Late Protogeometric	960 ± 30

**Fig 4:**
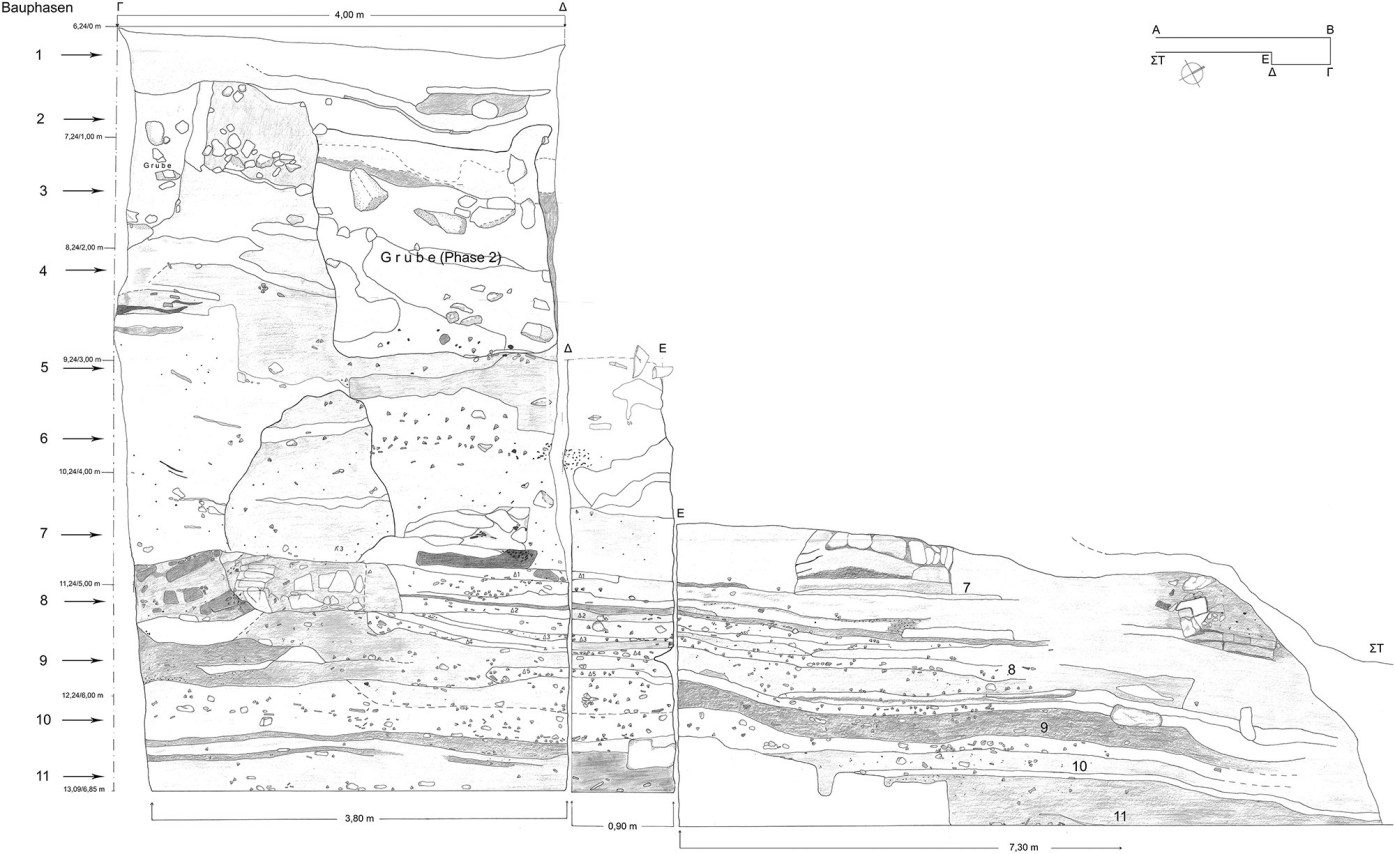
Eastern and southern profile of trench A.0 at Sindos.

Although a reconstruction of the Early Iron Age settlement plan is not possible at Sindos, as at few other Aegean Geometric sites such as Zagora, Sindos offers the unique opportunity for studying the Aegean material culture by means of a 13 m deep stratigraphy, which shows more than 16 settlement phases, at least 13 of which are successive (Fig 4). The large amount of pottery from its stratified contexts allows the comparative study of several Aegean pottery styles, in a region where pottery sequences of the Early Iron Age have until now been known almost exclusively by means of burial contexts. The finds from Sindos include non-local ceramic wares from many different Aegean micro-regions, such as Euboea, Attica and Corinth, and of course Macedonia. Regional correlations between the chronologies of the central and southern Aegean were already developed by Coldstream, as demonstrated in his most influential book on the Greek Geometric Pottery [15]. At that time any synchronization with the northern ‘periphery’ was still unthinkable. The Greek relative chronological system still suffers from all biases and ambiguities that resulted from its construction by means of burial contexts given that evidence from stratigraphies was practically missing. The fact that the inhabitants of this Aegean gateway to the Balkans made use of pottery from several parts of central and southern Greece makes Sindos a most appropriate place to achieve a major geographic expansion of the Aegean Early Iron Age relative chronology, and this now applies–due to its many Aegean contacts–to essentially the entire Mediterranean (see below).

An additional benefit of the long stratigraphy at Sindos is that it allows the newly developed Early Iron Age pottery chronology sequence to be dated by a series of N = 21 short-lived ^14^C-samples, that were all measured to high-precision (σ < 25 BP) by the Mannheim ^14^C-AMS laboratory (Lab-Code MAMS). The series covers essentially almost the entire cultural sequence of the Aegean Early Iron Age, beginning with Late Protogeometric, through Early Geometric I and II, Middle Geometric I and II, up to the Late Geometric Ia and Ib (Table 1).

All 21 samples of animal bone for the radiocarbon analysis were taken from good and relatively well-dated contexts at the stratigraphic trench A.0 that was excavated from 2000 to 2002 at the south-eastern side of the high table. The successive layers sloping to the south on this trench’s profiles reflect long process of debris accumulation at that side of the Early Iron Age table (Fig 4). While some of the earliest excavations at Sindos were conducted with the method of arbitrary layers, the most recent trenches including trench A.0 were excavated with the method of single context recording. Nevertheless, there is no archaeological procedure (known to us) to determine whether *bones* from allegedly ‘undisturbed’ contexts could be residual from earlier layers, or not. Such re-depositional processes may however be traced by means of *pottery*, especially in assemblages that have been statistically and typologically studied in such detail as those of Sindos. From these studies we know, for example, that the floors and contexts at trench A.0 of Sindos, where the animal bone samples were finally deposited, did not yield any noticeable residual ceramic or other artefacts.

The sampled bones of domestic animals (oxen/cows, pigs, sheep/goats) come from six successive phases of the settlement (phase 11 to 6) that date according to the conventional relative chronology from the Late Protogeometric (950–900 histBC) to the Late Geometric Ib (750–735 histBC). Note that samples were purposely also taken from phases 6, 7, and 8, despite the threatening expectation that their ^14^C-ages would have readings on the Hallstatt plateau (~800–400 calBC) of the ^14^C-age calibration curve.

The two latest samples were collected from the floor of a house of phase 6. Two of the six bones analysed from phase 7 were collected from the floor below the collapsed mudbrick wall and the roof of an earlier house. The other four bones of the same phase were found in contexts mixed with burnt material from the same house close to this wall. A thick layer of ash containing burnt clay probably from a house roof was covering the three bone samples together with other artefacts that were found on a floor of phase 8. Part of this destroyed settlement phase was levelled with debris that may have been brought from another part of the contemporary settlement. In any case no samples were collected from that context. The three bone samples from phase 9 were collected above the surface of a thin layer of yellow clay that used to cover the debris of an earlier phase and formed a new surface of use. All four bone samples of phase 10 were collected just above and within two successive thin layers of black earth that represent surfaces of use and relate to them. These were well-defined contexts that contained burnt material and large quantities of pottery and sea shells close to a wall that used to support a layer of yellow clay (terrace). The two sampled bones from phase 11 come from a similar context. This was a burnt layer with a lot of pottery and sea shells deposited on the surface of use, close to a massive and better preserved terrace wall. A more detailed description of the contexts of the bone samples can be taken from the section profiles and plans, which are available and analytically commented in the final publication of Sindos’ excavations [28].

To complete our brief review of the excavations, we note that the archaeozoological as well as archaeological material from the excavations at the settlement of Sindos is stored within the facilities of the Ephorate of the city of Thessaloniki. All necessary permits (ΥΠΟΠΑΙΘ/ΓΔΑΠΚ/ΔΣΑΝΜ/ΤΕΕ/Φ77/164609/3721) were obtained by the Greek Ministry of Culture for the described study, which complied with all relevant regulations.

### Introduction of the Sindos ^14^C-data

**Fig 5:**
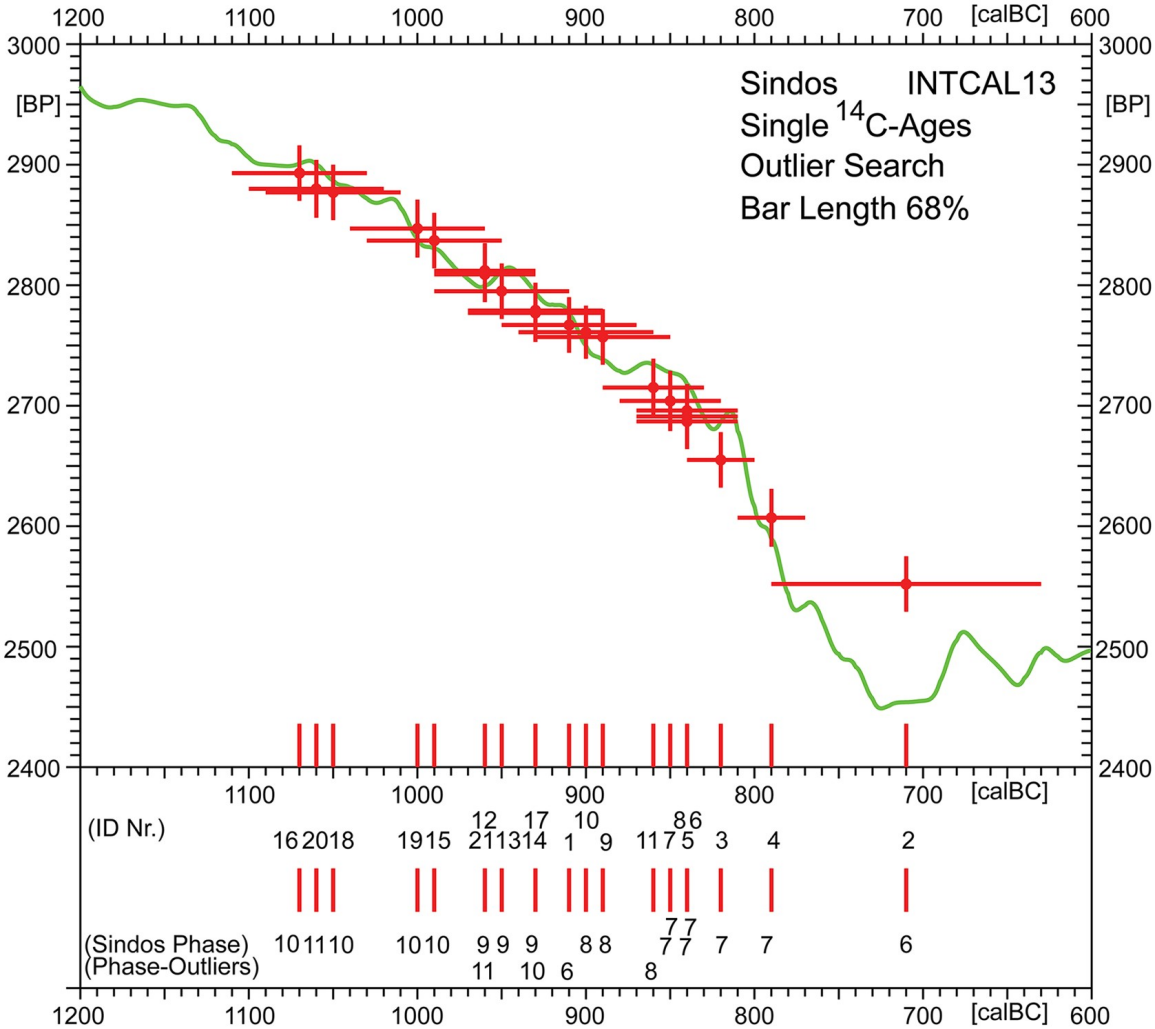
Overview of radiocarbon ages from Sindos (N = 21; Table 1).

Based on minimal age-modelling (i.e. albeit model-neutral, but not distortion-free; cf. below), Fig 5 provides an overview of the contents of the ^14^C-database (Table 1). In joint context with the INTCAL13-calibration curve and the high-precision laboratory data used in INTCAL-13 construction, Fig 5 shows the ^14^C-histogram and the summed calibrated ^14^C-age probability distribution of the summed Sindos data (N = 21), both in context with their individual BarCode-ages (small vertical lines on the calendric time-scale). The applied numbering of samples and Sindos-phases is useful for first screening of potential outliers (cf. Fig 5, lowest line), but this is more efficiently achieved by stratigraphic (metric) age-depth modelling (see below). The Barcode-ages are pragmatically defined as central values of the 95%-confidence intervals. For this kind of data-representation, aimed only on achieving a first summary of the overall data spread, it is important to note that the calendric-scale position of the barcodes is practically always strongly offset, in relation to the (unknown) calendric age. The horizontal age-distortion of single ^14^C-ages can be quite strong, in all variables (i.e. not only those evident in this graph), and is typically in the order of– 100 to + 100 yrs, but often more. This distortion is often only attributed to the existence of multiple calibration curve readings. However, from a more fundamental mathematical perspective, there are many such effects (including e.g. the decadal-scale clustering of Barcode-values, as can be taken from Fig 5), and all caused by one and the same factor, that is the non-commutative probabilistic algebra that is underlying the statistical properties of the ^14^C-age calibration curve. As goes for the present Sindos-series, the strongest age-distortion applies to the youngest date (ID2, MAMS-27019: 2552 ± 23 BP). This is easily recognisable, both from the extreme length of its error-bar as well as from the strong age-shift of the corresponding Barcode-line. Note that, in Fig 5, in order not to clutter up the picture, we have decided to show the error bars only at 68% confidence.

As goes for the Hallstatt Plateau (~800–400 calBC), an essentially flat region of the calibration curve that archaeologists prefer to avoid, it has its cause in the chance compensation of the increase in the natural atmospheric production of ^14^C and its decrease due to radioactive decay in parallel to its oceanic uptake. In consequence, when samples are taken from this time-window, by conventional wisdom all ^14^C-ages for the Middle Geometric II (800–760 histBC), Late Geometric Ia (760–750 hist BC) and Late Geometric Ib (750–735 histBC) would be expected to give the same ^14^C-age of 2480± 30 BP. As it turned out, none of the samples from Sindos have this ^14^C-content (Fig 5), with the exception of the obviously only quasi-distorted sample ID2, from the very youngest ^14^C-dated phase 6 at Sindos. Knowing that phase 6 follows immediately after phase 7, from the intercept of the ^14^C-age for ID2 with the INTCAL13 curve we can immediately provide a most precise (first) estimated reading of 780 ± 25 calBC for this phase. This is confirmed, later, by the explicit age-modelling (see below). Going back in time, all nine samples from phase 7 and phase 8 came from layers that were destroyed by fire, as evidenced by the large amounts of ashes, charcoal, and collapsed mud brick walls on the settlements floors. Even such apparently ‘safe’ contexts, however, do not entirely eliminate the possibility that some of the animals, whose bones were deposited in these two (or any of the other settlement phases), may actually have been consumed in an earlier phase. There are however reasons to think that such biases are less possible to have affected our sampling (see above).

### Stratigraphic ^14^C-based age-depth modelling at Sindos

**Fig 6:**
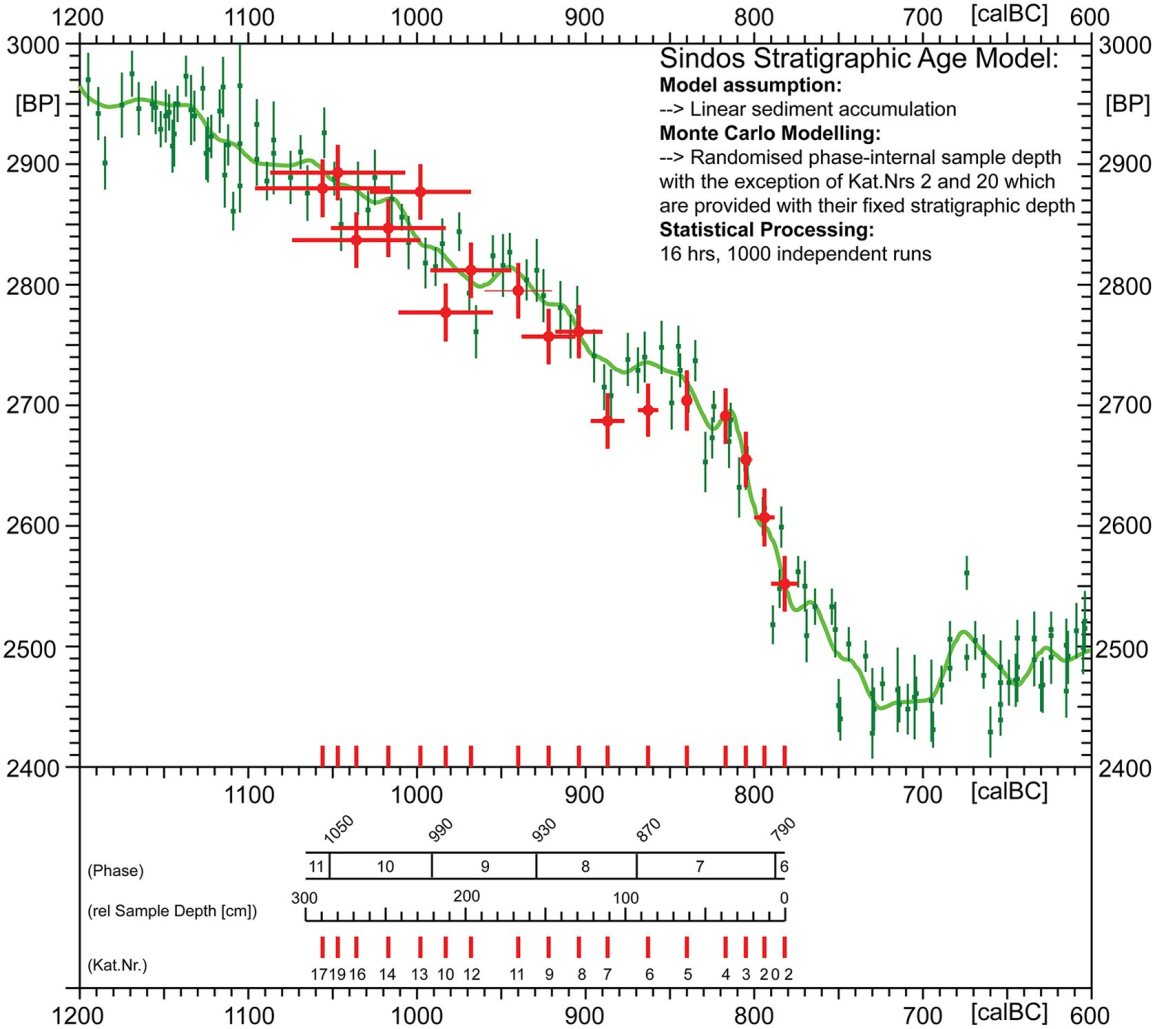
Chronological results for Sindos (phase 11–6) achieved by application of Gaussian Monte Carlo Wiggle Matching (GMCWM) to N = 17 stratigraphically screened ^14^C-ages.

**Fig 7:**
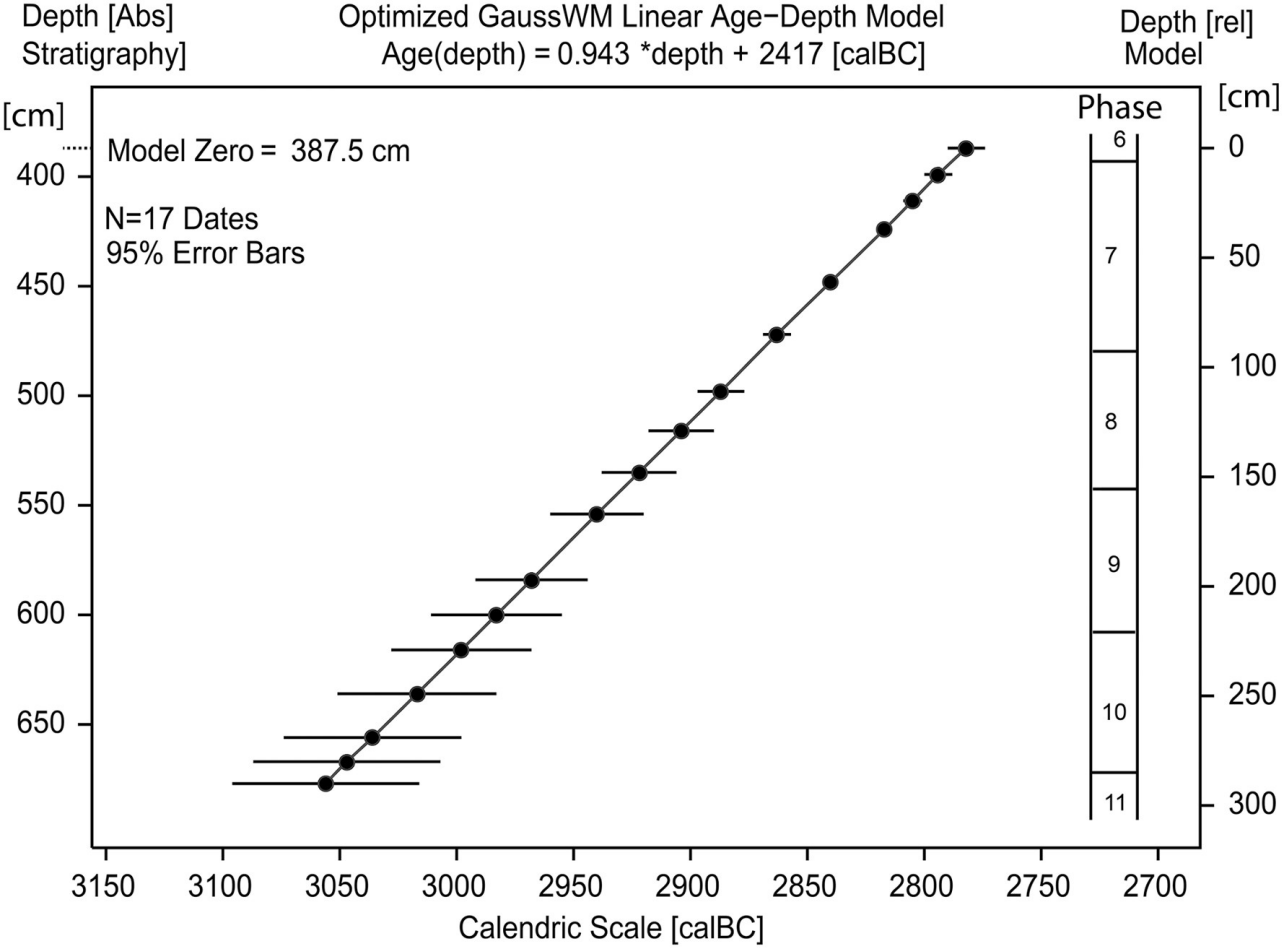
Monte-Carlo-based dating errors derived from the age-depth model.

For age-depth modelling at Sindos we have applied the method of Gaussian Monte Carlo Wiggle Matching (GMCWM *or* GaussWM) [2]. The achieved chronological results are shown in Fig 6, with corresponding modelling uncertainties shown in Fig 7. The numeric modelling values are provided in Table 2. Before continuing with the archaeological discussion of these results, let us take a moment to evaluate the applied method itself. In historical perspective, GMWCM is an extended and today largely automated version of the wiggle-matching method that was first introduced in the year 1986 by Gordon Pearson in support of the (preliminary) dating of floating tree-ring chronologies based on matching a series of ^14^C-ages to the calibration curve [32]. Also in 1986 essentially the same (Chi-squared) method was applied to stratified ^14^C-ages from Tell Dipsis (Bulgaria), Niederwil (Switzerland) and Arslantepe (Turkey), as well as to historically seriated ^14^C-data from the Egyptian 1^st^ Dynasty [33]. An important drawback of these earliest applications, however, was the difficulty in determining the statistical uncertainties of the dating results, but which can be overcome by including a Monte-Carlo simulation of the different error sources [34]. Hence, despite a steadily increasing number of extensions and modifications applied over the years (e.g. [1] [2] [3] [34]), the GMWCM-procedure is still today based on essentially the same method as it was, some 30 years ago. As illustrated (schematically) in Eq (1), the approach taken is to minimize the statistical distance (on the ^14^C-scale) between the discretely measured sequence of tree-ring (or archaeological) samples that have ^14^C-ages D_i_ ± σ(D)_i_ [BP], but unknown calendric ages, and the continuous (e.g. splined) calibration curve that has ^14^C-ages K(t) ± σ(K)_i_ [BP]) at certain known-age calendric years t.

$$\chi^{2}=\sum_{\text{i}=1}^{\text{n}}\frac{\left({\text{D}}_{\text{i}}-\text{K}{\left(\text{t}\right)}_{\text{i}}^{2}\right)}{\left(\sigma {\left(\text{D}\right)}_{\text{i}}^{2}+\sigma {\left(\text{K}\right)}_{\text{i}}^{2}\right)}$$ (1)

**Table 2:** Data input and results for GaussWM-analysis of stratified ^14^C-ages from Sindos.

Kat Nr	Lab Code	^14^C-Age [BP]±1σ	Depth [m]	Sindos Phase	Random Depth [m] Position	Random Age [a] Position	Result [calBC] 95%	Conventional Age [BC]
Kat.Nr.2	Hd-27019	2552 ± 23	3,875	6	3,875 m	±10a	782 ± 8	750–735
Kat.Nr.20	Hd-27037	2607 ± 24	3,995	7	3,995–4,140 m	±10a	794 ± 6	760–750
Kat.Nr.3	Hd-27020	2655 ± 23	4,140	7	3,995–4,140 m	±10a	805 ± 4	760–750
Kat.Nr.4	Hd-27022	2691 ± 23	4,140	7	3,995–4,140 m	±10a	817 ± 2	760–750
Kat.Nr.5	Hd-27023	2704 ± 25	4,140	7	3,995–4,140 m	±10a	840 ± 2	760–750
Kat.Nr.6	Hd-27024	2696 ± 22	4,140	7	3,995–4,140 m	±10a	863 ± 6	760–750
Kat.Nr.7	Hd-27021	2687 ± 23	4,140	7	3,995–4,140 m	±10a	887 ± 10	760–750
Kat.Nr.8	Hd-27026	2761 ± 22	5,005	8	5,005 m	±10a	904 ± 14	800–760
Kat.Nr.9	Hd-27025	2757 ± 23	5,005	8	5,005 m	±10a	922 ± 16	800–760
Kat.Nr.11	Hd-27028	2795 ± 23	5,560	9	5,560–5,785 m	±10a	940 ± 20	850–800
Kat.Nr.12	Hd-27027	2812 ± 23	5,560	9	5,560–5,785 m	±10a	968 ± 24	850–800
Kat.Nr.10	Hd-27029	2777 ± 24	5,785	9	5,560–5,785 m	±10a	983 ± 28	850–800
Kat.Nr.13	Hd-27033	2877 ± 23	6,175	10	6,175–6,300 m	±10a	998 ± 30	900–850
Kat.Nr.14	Hd-27036	2847 ± 24	6,175	10	6,175–6,300 m	±10a	1017 ± 34	900–850
Kat.Nr.16	Hd-27030	2837 ± 23	6,300	10	6,175–6,300 m	±10a	1036 ± 38	900–850
Kat.Nr.19	Hd-27031	2893 ± 23	6,300	10	6,175–6,300 m	±10a	1047 ± 40	900–850
Kat.Nr.17	Hd-27034	2880 ± 24	6,790	11	6,790 m	±10a	1056 ± 40	950–900

The samples are arranged in stratigraphic order, with stratigraphically highest (‘youngest’) sample (Hd-27019) at table top, and stratigraphically lowest (‘oldest’) sample (Hd-27034) at table bottom. The Monte Carlo results are provided with measured marginal uncertainties (noted at 95%-confidence), without numeric rounding. During run-time, a Gaussian-shaped random error of ± 10 [a] was added to the numeric age of each sample derived from the (itself randomised) depth-position. The intention hereby is to include not only (possible) phase-internal but also phase-overlapping sample re-location. Note that, due to the Monte Carlo procedures, the overall dating results are given, not for each sample (*per se*) but for the associated stratigraphic sample position. Further quadratic addition of 20 yrs error (95%-confidence) to each of these stratigraphic positions would appear advisable, to account for unrecognised error components, but which cannot be proven to exist.

Accordingly, there exists a statistically best-fitting year t, not only for ^14^C-ages measured on tree-ring series (as in the application by Gordon Pearson), but similarly for many other kinds of seriated, sequenced, or otherwise stratified sample series. All that is needed is that the series has a temporal structure (call it an ‘age model’). Potential applications for the required age-modelling can be based on known (metric-scale) historical time-spans, as for the Egyptian historical chronology mentioned above, but also on sequences of ordinal-scale (older/younger) settlement phases. The method is also applicable to archaeological tell-stratigraphies, as in the present paper, in which case the age-modelling can be based on the measured (metric-scale) stratigraphic sample depth. The special advantage of applying the χ2-method to metric-scaled sample sequences is that the required numeric modelling values are immediately available (as measured depths) and forthwith only require rescaling (depth->age). Of course, in practical applications, the necessary depth->age rescaling requires a fair amount of statistical processing (cf. below). But, from a mathematical viewpoint, there are only minor differences between the different Chi-squared approaches. For example, whereas in dendro-studies the distances between the ^14^C-dated (annual growth) samples have small sampling errors (0–2 yrs), in archaeological studies the distances between dated samples can be quite large (for settlement phases: 10–50 yrs). This makes the necessary modelling estimates, in archaeological studies, inherently much more error-prone than in the dendrochronological application. In consequence, although archaeological GMWCM-studies require no fundamental change in the mathematical algorithms, they do require further attention in terms of error analysis. Next to such technical aspects, the really important advantage of applying GMWCM to tell-stratigraphies (e.g. Sindos) is that the validity of the modelling assumptions can be checked, namely, by direct comparison of the model-ages achieved for each sample, with the results based on the respective (unmodelled) single calibrated ^14^C-ages. This also applies *vice-versa*.

Nevertheless, although in concept simple, the advantages of metric age-modelling are not received for free. Rather more, the application of metric modelling to real archaeological data is immediately complicated by the need for more advanced (and higher quality-level) requirements already during archaeological sampling, and similarly during the statistical data processing, hereby in terms of data input/output procedures, age-modelling algorithms, application of randomization requirements, calculation of probabilities, and graphic output routines. In the course of the methodological extensions of the GMCWM-method described in [29] [34], today the technical procedures are largely automated.

**Fig 8:**
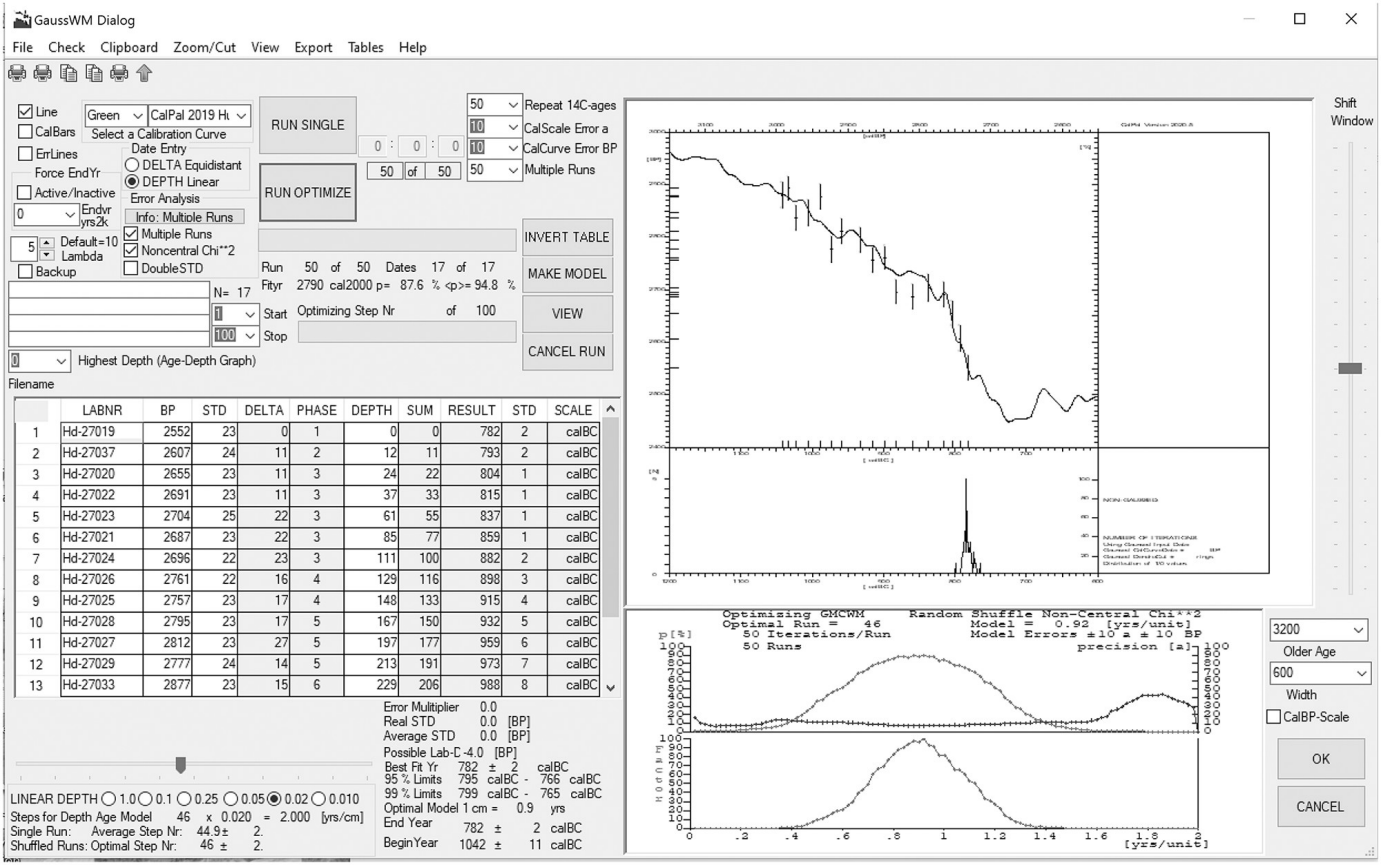
GaussWM dialog of CalPal-software (version 2020.2), illustrating the stratigraphic age-modelling analysis of Sindos ^14^C-data. *Left*: spreadsheet with input of data and results; program functions; tools for age-model construction; tools for statistical analysis. *Right Upper*: Graph showing on-screen (Monte Carlo run-time animation) the presently achieved best-fit position of the archaeological ^14^C-series in context with the selected calibration curve. *Right Middle*: Histogram showing the dating probability and precision of the step-wise expanded ^14^C-series. *Right Lower*: Statistical fit parameters are given as run-time series of the stepwise expanded sequence. Red = dating probability; Blue = dating precision; Green = simultaneously optimized precision and probability. Note: During run-time the GaussWM tables and graphs are continually refreshed (every ~3 secs). An explorative model analysis requires ca. 1–5 min run-time. Typical runs require 6–16 hours.

The application of GMCWM to the Sindos ^14^C-series is illustrated by a screenshot (Fig 8) of the most recent GaussWM-dialog, which is integrated in CalPal-software (Version 2020.2). In terms of hardware, the results were obtained using workstation Celsius W530^®^ with Xeon E3-1281v3^®^ 3.7 GHz CPU. In terms of software, CalPal is written in programming language Fortran 95, with compilation by Intel^®^ Parallel Studio XE Fortran Compiler, in combination with Winteracter^®^13 and IMSL6.0^®^ libraries. Data transfer in Excel^©^-format is achieved through the ODBC^©^-Interface (Open Database Connectivity). Such ODBC-compliancy greatly simplifies the analysis of archaeological ^14^C-data, due to the possibility of user-convenient external data-editing.

An important amendment of the GMCWM procedure, used in the Sindos-analysis, is the application of a refined data/curve fitting method, based on the non-central Chi-squared distribution instead of Chi-squared. This does not change the GMCWM results, but makes the analysis less sensitive to slightly asymmetric ^14^C-data, as may be caused by chance, or small (decadel-scale) ^14^C-reservoir deviations and interlaboratory offsets. The use of non-central Chi-squared probabilities require calculations of the incomplete Gamma function, which were conveniently performed (in Fortran 95) by a call to the IMSL^®^-subroutine CSNDF. The advantage of using CSNDF, in comparison to GAMMQ, is that allows passing of a variable noncentrality parameter λ. In the Sindos analysis we applied λ = 5 (for λ = 0 CSNDF converges to a Chi-squared test). As shown schematically in Eq (2)) the calculation of best-fitting probabilities based on CSNDF follows directly after the Chi-squared distance calculations.

$$\begin{array}{lll} \text{P}\left(\chi^{2},v,\lambda \right)\to & \left(\text{IMSL}\;\text{Fortran}\;\text{Library}:\text{CSNDF}\right)\to & \text{Gamma}\;\text{Function}\\ \text{Moncentral}\;\chi^{2} & v=\text{Degrees}\;\text{of}\;\text{Fredom} & \\ \text{Probability} & \lambda =\text{Noncentrality}\;\text{Parameter} & \end{array}$$ (2)

#### Run time parameters

The final Sindos results are based on a GMCWM run with the following parameters and program settings. The model was run for a total of 1000*50*60 = 3*10^6^ random iterations. These included, (1) a fixed number of 60 steps in the sequence expansion, (2) a number of 50 Monte Carlo iterations for phase-internal re-ordering, and (3) a total number of 1000 complete run repetitions. In effect, therefore, the modelling results shown in Table 2 (Column: *Results*) are based on 1000 independent measurements., each of which was obtained as best-result of an extended search. During run-time, in parallel to the phase-internal randomized sample order (Table 2, Column: *Random Depth Position*), the sample distances were also randomized, with additional (squared additive) Gaussian calendric-scale distance errors set to σ = 10 yrs (Table 2, Column: *Random Age*). Finally, as a final error component, applied prior to each model-expansion, the calibration curve (INTCAL13) was re-splined, with the new curve in each case based on the original INTCAL13 raw data, but re-measured with Gaussian ^14^C-scale errors set to σ = 10 BP. The run-time of the final Sindos age-modelling run was 16 hours.

In a nutshell, the best-fitting position of the Sindos ^14^C-data series on the INCAL13 calibration curve (shown in Fig 6) was identified by systematic stepwise linear-expansion of the sample sequence. The applied linear-stratigraphic age-model was constructed according to the measured sample depth. The validity of this age-model, with modelling errors in der of range of 10–20 yrs (95% confidence), is confirmed due to the reproducibility of the chronological results when only the single ^14^C-ages are used i.e our interpretations are altogether independent of the assumptions (whether critical or not) that are at the base of the age-depth modelling. The modelling results are nonetheless useful. They allow the derivation of a simple linear equation, as shown in Fig 7, that conveniently supports the dating (with associated errors) for any requested tell-depth.

Although the main aim of GMCWM in stratigraphic studies is, naturally, to identify the best-fitting length and age-position of the ^14^C-dated sample sequence on the ^14^C-age calibration curve (here: INTCAL13), what is actually challenging–as mentioned above—is the derivation of (albeit) realistic dating errors. Of special interest, in this respect, is to derive the marginal probabilities that are assigned (here) to the different Sindos phases. In the present GMWCM–application to the Sindos data, as it turns out, the (calculated) marginal dating uncertainties are quite small (annual-scale) for the youngest phases (7 and 8), but increase strongly for the older phases (Fig 7). Understandably, this is the immediate consequence of the shape of the calibration curve in the time-window under study (1100–700 calBC), which shows a rather wiggly section for ages 1100–800 calBC, followed by a major increase in slope, if only for some 20 yrs (~800–780 calBC). Lucky are those archaeologists, in terms of achievable supra-precision, whose ^14^C-dated samples have readings into this time-window. Yet, this time-window–wide as it may appear (Fig 5)–is narrower than it looks (from a statistical perspective), as well as representing a most strongly wobbling target (from the view-point of tell-related sedimentation processes).

Note that, with this intention, we must foremost (quantitatively) allow for possible stratigraphic reworking of samples. In the present application, this is attempted both by controlled *intra-phase* Monte Carlo randomization of the stratigraphic sample position (for multiple-dated phases i.e. phases 7–10), as well as by Gaussian *inter-phase* spreading of samples, for all phases. Whereas the Monte Carlo randomization is achieved by application of a random-position algorithm only to samples from the multiple-dated phases, the Gaussian sample spreading is achieved by applying an additional (depth-controlled) Gaussian age-distribution, with chosen width of ± 10 yrs (68% confidence) to all sample depths. Even under such, at least, intentionally realistic Monte Carlo conditions, what we actually observe for the younger phases are calculated dating uncertainties smaller than 10 yrs. Given that ^14^C-AMS interlaboratory offsets are presently estimated to have values of ± 10 BP, at best, we have accordingly enlarged the calculated errors to this value (on the calendric time scale: ± 10 yrs) for all phases. Nonetheless, Figs 6 and 7 show the original (uncorrected) uncertainty values, if only for purposes of critical interrogation. Put differently, we believe we have the reserve in dating caution that is necessary for targeting the window of supra-high dating precision, as noted above. However, what is equally if not more critical to demonstrate is the stratigraphic integrity of the ^14^C-dated samples, and this in combination with the archaeological finds. To this aim, on the following, we provide a detailed archaeological description of the site. Our focus is on the pottery inventory, which is to be used for wider synchronisms, and which is presented in a phase-by-phase manner, from old to young.

### Relative pottery-based chronology at Sindos

The relative chronology of the stratigraphic sequence at Sindos was achieved after some quite exhaustive statistical, typological and technological analysis of its large pottery assemblage that comprised 4897 rim-sherds and numerous other wall and other fragments from well-stratified contexts. The study of pottery technology took place independently from its typological analysis. By means of a x40 stereoscope pottery fabrics were macroscopically described and classified in 32 major ceramic ware groups and further subgroups (plain handmade wares not included). Macroscopic fabric descriptions and ware characterisations were supported by an extensive Neutron Activations Analysis project that was conducted in cooperation with Hans Mommsen (forthcoming). The typological study of the ceramic material of Sindos resulted after detailed contextual analysis and cross-comparison with other contexts in the Aegean and Mediterranean, where pottery shapes and fabrics of the same type as those at Sindos were also in use. Finally, statistical analysis based exclusively on rim sherd count of 4897 fragments from the best stratified settlement contexts [28] [35].

Well-dated, non-local pottery facilitated correlation with other regional chronological systems in the Aegean, which was further achieved through analytical studies and cross-checking of local ceramic types. The origin of the local and non-local (mainly Euboean and Attic) pottery types that were used at Sindos has been scientifically defined by means of Neutron Activation Analysis of a representative pottery sample (see above). The non-local pottery used in certain settlement phases comprised a considerable part of the total ceramic assemblage consumed at the site. In particular, 5% of the total pottery in the settlement phase 8 was not local; in phases 7 and 6 the rate was 7% and 9% respectively. The majority of the non-local pottery at Sindos came from Euboea: 92% of the imported wares from phase 7 (169 individuals) came from that island [28]. The use of such large quantities of non-local pottery can barely support its perception as commodity of particular symbolic or other value that may have remained in use for some considerably longer period of time than it did in its place of origin/production. The assumption of overall short-use is further supported by the fact that broken vessels of non-local origin were never repaired–as otherwise usually happened with similar wares in other non-Greek contexts, where bore holes are common on Greek pots–but were immediately rejected. Finally, most of the non-local pots do not show traces of intense use such as chipping and wearing of the paint. It is thus reasonable to assume that the time from production to final deposition of these pots was not considerably different between the place of origin and place of consumption.

In the lowest ^14^C-dated phase 11 at Sindos, for the first time in the stratigraphy, we find pottery sherds of wheel-made vases with concentric semicircles. At Kastanas and Lefkandi, this motive appears for the first time during the Middle Protogeometric period. Nonetheless, phase 11 of Sindos probably does not date that early, since a skyphos fragment with a group of zig-zag lines in the handle zone from this phase has numerous parallels in phase 10 at Kastanas that has been firmly dated to Late Protogeometric [28] [36].

The pottery from the immediately following phase 10 of Sindos points to a correlation with the Early Geometric or Subprotogeometric I–II periods in southern and central Greece. In this settlement phase appear at Sindos for the first time–at least in considerable numbers–the Thessalo-macedonian cantharoi of type I. This phase has also yielded the earliest fragments of pendent semicircle skyphoi at Sindos as well as numerous fragments of northern Aegean Transport Amphoras of transitional type. Although production of most of these pottery types began in the Late Protogeometric, and continued into the next period, phase 10 can be dated in the Early Geometric or Subprotogeometric I–II phases, i.e. in the first half of the 9^th^ century according to the conventional chronology, by means of a sherd from a bowl with offset rim that finds good parallels in a closed burial context of the Subprotogeometric I–II at Lefkandi according to the local ceramic sequence [28].

The overall picture for the ceramic assemblage of phase 9 leaves no doubt that its wheel- and handmade pottery belongs to a period earlier than Middle Geometric II. Especially two ceramic sherds from closed contexts offer a firm date in Middle Geometric I/Subprotogeometric IIIa. On the one hand, the terminus ante quem is set by a fragment of a pendent semicircle skyphos of type 2, which was not produced any more after the end of the Middle Geometric I/Subprotogeometric period IIIa. The terminus post quem for the date of phase 9, on the other hand, is offered by a sherd of Euboean crater with monochrome conical foot decorated with horizontal bulges. This fragment comes from a crater of type II, which cannot be earlier than Middle Geometric I/Subprotogeometric IIIa.

In settlement phase 8 considerable quantities of imported ceramic wares were now used at Sindos for the first time. The most common non-local wares were pendent semicircle skyphoi of the types 4 and 5 that are usually dated in Middle Geometric II, i.e. first half of the 8^th^ century according to the conventional chronology. This relative chronology of phase 8 is confirmed by several other imported Euboean and Attic wares, which cannot typologically date before Middle Geometric II or after the beginning of Late Geometric. Buildings and other structures of the two best pottery-dated phases predating the Late Geometric phase 7 were excavated on the upper as well as lower table of the settlement [28].

**Fig 9:**
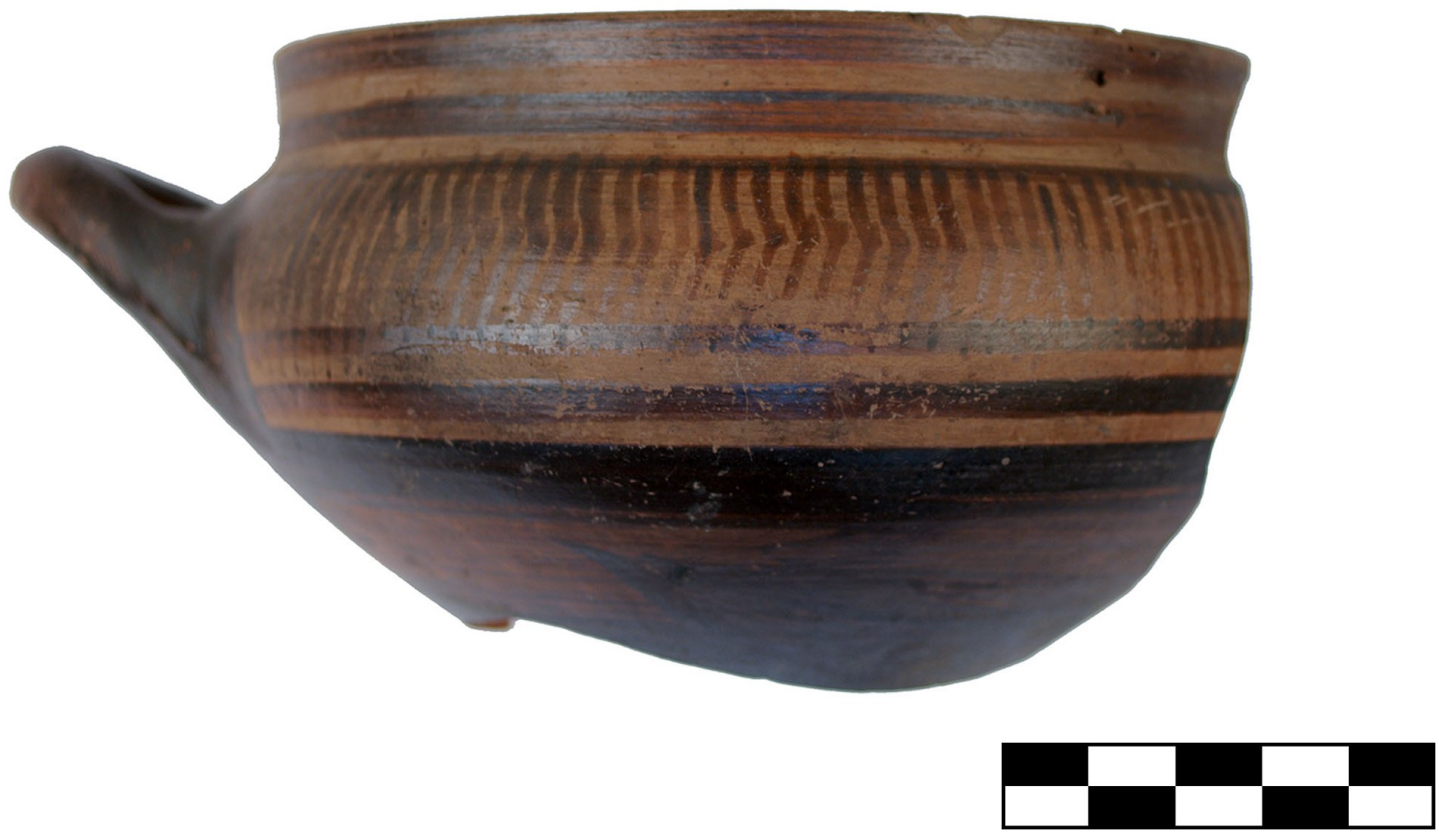
Euboean skyphos with chevrons from phase 7 at Sindos (analysed with Neutron Activation).

**Fig 10:**
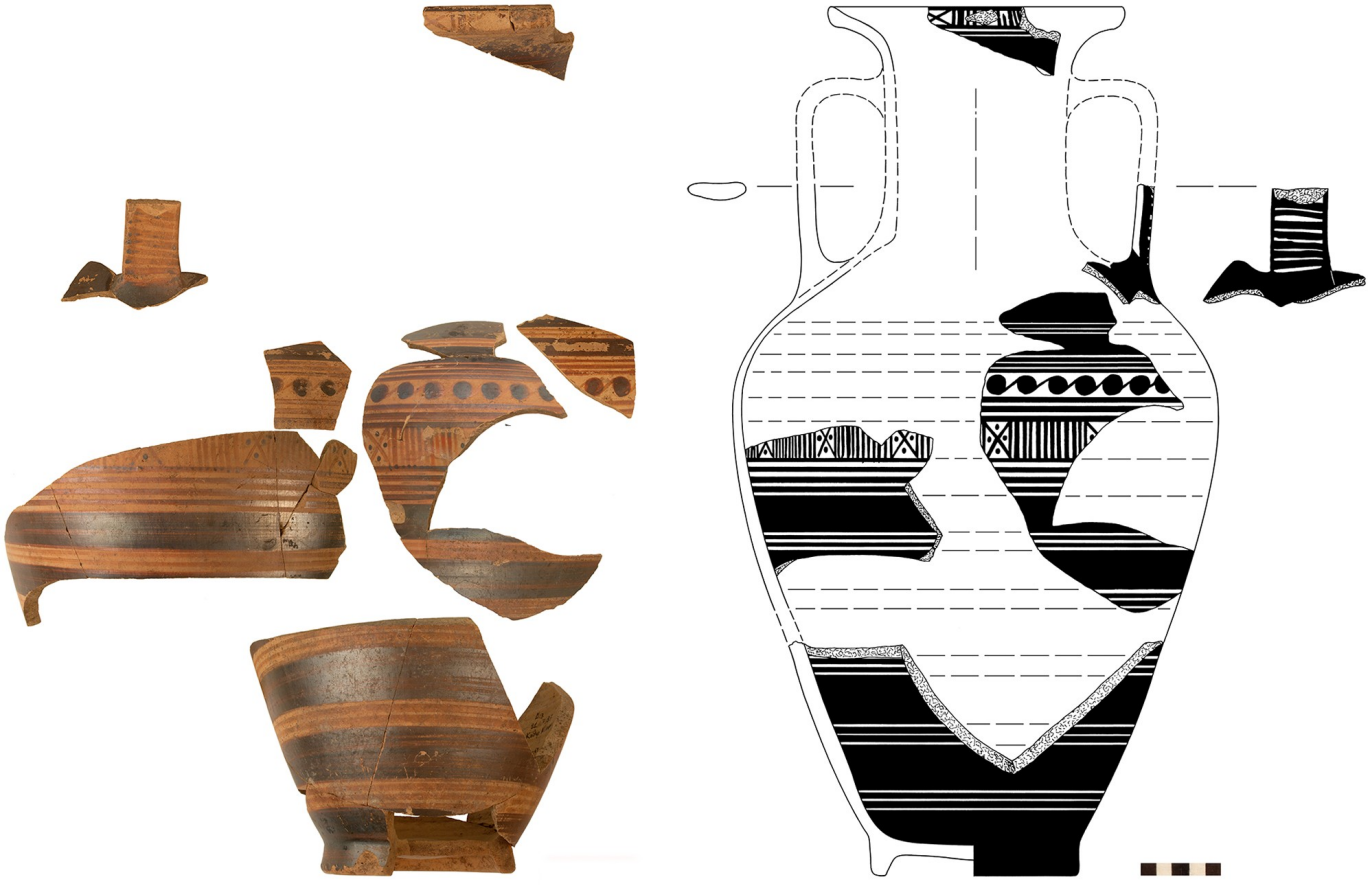
Attic amphora of Late Geometric Ia from phase 7 at Sindos (analysed with Neutron Activation).

With phase 7 the settlement of Sindos reached its largest extent (5 ha), and also experienced some remarkable transformations in material culture, including significant innovations in its pottery technology and consumption [35]. Phase 7 is securely dated by means of Attic and Euboean pottery to Late Geometric Ia, a chronological sub-period that allegedly occupied a single decade, which would make it by far the shortest settlement phase at Sindos, according to the conventional chronology [28]. During phase 7 deep Euboean skyphoi decorated with concentric circles, dashes and other linear motives on the high lip as well as panels with meanders or hooks and chevrons (Fig 9) or metopes with birds and quatrefoils in the handle zone–all of which are typical of the Late Geometric I period–appeared for the first time at Sindos. At the same time some very characteristic and well-dated Attic vases of Late Geometric Ia with exact typological parallels in the well-defined seriations of Attic pottery were also imported and used at Sindos (Fig 10).

After the destruction of phase 7 by fire, the lower table was not occupied again until the middle of the 6^th^ century BC, according to the conventional Aegean chronology. The new settlement of phase 6 was now restricted to the smaller plateau of the higher table. The datable pottery of this phase comes mostly from Euboea and continues the previous tradition of the deep skyphoi with the characteristic motives on the lip and the decoration of the handle zone with metopes and panels with hatched hooks and meanders that disappeared only in Late Geometric II. This settlement phase has been subsequently dated to the sub-period Late Geometric Ib, which was defined in Athens by means of a sequence of burial contexts in combination with typological criteria. The next two habitation phases at Sindos, which both predate the 7^th^ and early 6^th^ century hiatus, both belong to the Late Geometric period. Their pottery shows features of the later typological developments in the Euboean ceramic sequence that leave no doubt for an assignment to Late Geometric II [28].

### Implications of Sindos stratigraphy for the Greek relative chronology of the Early Iron Age

The study of the pottery finds at Sindos has strongly enhanced our understanding of the temporal changes in ceramic styles not only in the northern but also in the central Aegean, especially during the Geometric period [28]. Already for this period, there are eight successive phases at Sindos, two of which–phases 7 and 8 –have been excavated to some large extent. Thanks to the continuous stratigraphy of Sindos, it was possible to elucidate for the first time the typo-chronological development of local ceramic categories such as the Protogeometric and Geometric Transport Amphoras and the K 22-Ware as well as certain categories of Euboean wares, such the pendent semicircle skyphoi, the chevron skyphoi (Fig 9) and other types of Atticising and Euboeaning Geometric pottery.

Another significant outcome of pottery studies at Sindos is that certain ceramic wares, previously identified as hallmarks of the pottery production at microregions in central Greece such as Euboea were apparently also locally produced in Macedonia. What is also important, the Macedonian pottery production does not simply imitate the allegedly innovative pottery styles of these regions, which are usually perceived as ‘centres’ with more complex social and economic organisation. Even the local Macedonian wares were part of a common pottery tradition that was dominant everywhere in the north-western Aegean, from Chalkidike and central Macedonia to eastern Thessaly, Phthiotis, Euboea and northern Cyclades [37]. We recognise that, for example, locally produced skyphoi with pendent semicircles of the same types were produced and used at the same time in Macedonia and Euboea. In clear contradiction to earlier art historical conceptualisations of ancient pottery production and exchange, the recent typo-chronological analysis of the pendent semicircle skyphoi, based on the new finds from Sindos and other sites in central and western Macedonia, shows that the invention of this type in Euboea should not be taken for granted. We should thus keep distance from views that regard the production of many well-known ceramic types in the Aegean ‘periphery’ as later adaptions to stylistic innovations that originated at certain ‘centres’. The use of essentially identical pottery types of both Euboean and Macedonian origin took place, as it now appears, at the same time at Sindos. This conclusion is significant for the purposes of the present study since it allows to argue also by means of local pottery for the correlation of northern and central Aegean relative chronological systems [28].

It would be fair to state that the stratigraphy of Sindos has proven as helpful for our comprehension of the temporal development of the many non-local ceramic wares, as the excavations conducted at their assumed place of origin. This is true for certain categories of Euboean Middle and Late Geometric pottery that were widely circulating and used in other regions of the Mediterranean, the typological development of which was a much-disputed topic. The most recent excavations at Eretria and their subsequent publication have added much knowledge for the typological development of these wares, but a detailed pottery sequence is still missing in Euboea itself, since the Eretrian pottery contexts are only broadly datable into two or more chronological periods [38] [39]. The fine stratigraphy at Sindos provided more detailed information concerning the typological development of Euboean pottery, especially during Middle and Late Geometric [28].

There are two main reasons that Euboean and Corinthian pottery of the Geometric period has attracted so much scholarly interest in the past decades: first, these are some of the earliest Aegean wares that were circulated and massively used in the Mediterranean after a long break following the end of the Late Bronze Age. Second, the same wares provide what is perceived through a culture-historical perspective as hard evidence for the Greek colonisation.

Even when viewed from the traditional centre-periphery perspective that has dominated historical interpretations for the last two centuries, Sindos offers detailed data not only for the ‘regional’ Macedonian pottery sequence, but also to comprehend the developments at certain ‘centres’ of the central and southern Aegean. In conclusion, due to its long and continuous stratigraphic sequence and its potential for both long- and short-distance correlation of pottery styles the site of Sindos is one of the few places in the Aegean, where it is possible to test the historical chronology of the Early Iron Age. In the present paper this is now accomplished by a series of radiocarbon dates that were measured on a long stratigraphic sequence of short-lived bone samples.

## Discussion

### Revision of the Greek Early Iron Age chronology by means of the new radiocarbon dates from Sindos

**Fig 11:**
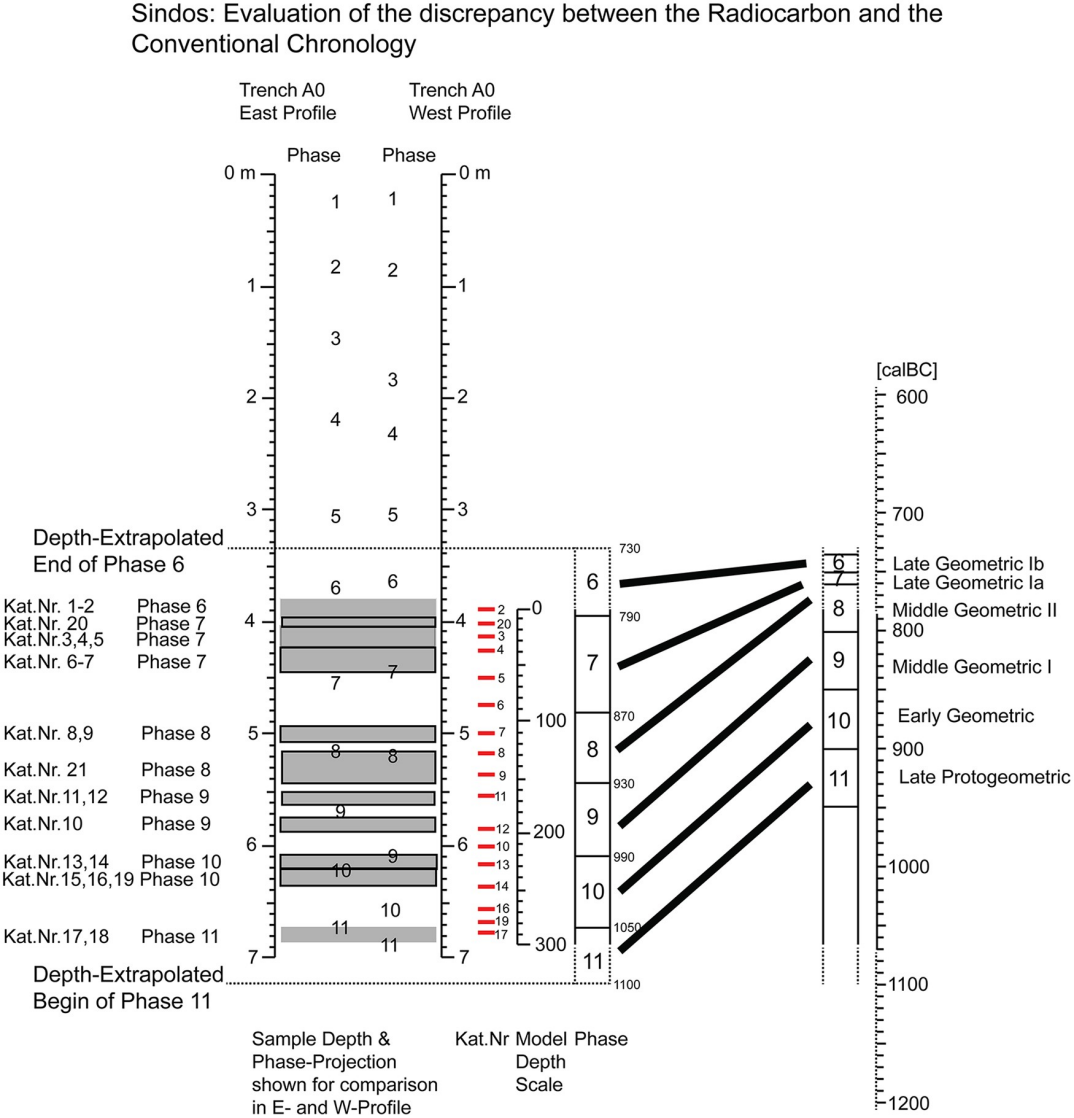
Comparison of absolute and relative chronologies: Sindos (left) vs historical (right).

The new radiocarbon dates from Sindos have important implications for the Greek Early Iron Age chronology, in particular for the periods older than Late Geometric Ib. The dates for the end of the Late Geometric and the beginning of the Archaic period are less affected. A comparison of the newly achieved ^14^C-based absolute chronology from Sindos with the conventional chronology is provided in Fig 11.

It is noteworthy that the traditional sequence of Protogeometric and Geometric periods is well-confirmed by the stratified ^14^C-ages from Sindos. Already the satisfactory coherence of the relative pottery sequence, as established at Sindos in high stratigraphic resolution, speaks for the wider applicability of the new chronology. This, of course, requires further confirmation.

The first major implication of the new chronology relates to our understanding of the Late Geometric I period, which is usually perceived as a transformative phase not only for Greece but also for the Mediterranean through an intensification of contacts between the Aegean and the Levant, and the beginning of Greek ‘colonial’ expansion towards the West. All these events are thought to have taken place within a single generation, from 760 to 735 BC (see analytically above). Eight ^14^C determinations from phases 6 and 7 at Sindos dating in Late Geometric Ib and Ia respectively present new solid evidence for a redefinition of this historically significant phase. Hints for a higher chronology of the Late Geometric I period from problematic contexts in central and western Mediterranean such as those of Carthage and others, where random Geometric pottery sherds were found [40] [41], are confirmed by the new ^14^C-series from the northern Aegean, which show that this period may have been longer than usually thought. At Sindos it has empirically measured time span of ca. 130/140 years. Interestingly, the limit between Late Geometric Ia and Ib is now set at 790 calBC and beginning of Late Geometric I at 870 calBC. Settlement phase 7 of Sindos, which dates to the allegedly only short period of Late Geometric Ia, is the best sampled (N = 6 ^14^C-ages) settlement phase of the entire sequence. In addition, since the ^14^C-ages from phase 7 reach well into the steep section of the calibration curve, Late Geometric Ia is also the most precisely dated period at Sindos. In consequence, Late Geometric Ia should not be taken any more as the shortest chronological phase of the Greek Early Iron Age as originally perceived by Coldstream. It covers a time-span much longer than that of other Geometric periods (Fig 11), just as suggested based on the many discernible cultural changes and innovations that took place during this period, all around the Mediterranean.

Three ^14^C-determinations from phase 8 raise the beginning of Middle Geometric II from 800 histBC to 930 calBC, a result which agrees well with the recently published radiocarbon dates for seeds from a secure context at Utica in Tunisia, which contained plentiful amounts of Middle Geometric II pottery [42].

Three further ^14^C-ages from settlement phase 9 place Middle Geometric I well into the 10th century, while five ^14^C determinations from phase 10 may raise the Early Geometric period–again by around 100 to 150 years–into the second half of the 11th century BC.

Finally, two radiocarbon dates from phase 11 at Sindos that probably dates to Late Protogeometric seem to place this phase of the relative Greek chronology into the first half of the 11th century BC.

Due to missing scientific evidence we may only assume that the underlying Early and Middle Protogeometric would date somewhere before the end of the 12th and beginning of the 11th century BC. To this point it is important to remember the conjectural character of the tripartite definition of the Protogeometric period. Strongly in need of clarification, in relative terms, is the definition of its Middle phase, which is exclusively based on some few mortuary contexts in combination with some not well-defined typological sequences in central and southern Greece. It is mainly for these reasons that the Middle Protogeometric is an extremely elusive phase, especially within settlement contexts.

Sindos is the first Aegean site for which a continuous sequence of ^14^C-ages on short-lived samples (animal bones) from the Early Iron Age is now available. Its main amenity is to support critical evaluation of the orthodox historical chronology for the Protogeometric and Geometric periods in north, central and southern Greece. Classical archaeology has long relished a quite unique privilege throughout the circum-Mediterranean chronological systems, in that the underlying absolute dates are based on comprehensive faith in the validity of the antique historiography. The proposed revision of the Greek Early Iron Age (absolute) chronology is in accordance with previous analyses and studies in the northern Aegean as well as in the eastern and western Mediterranean that support a higher chronology [4] [9] [40] [41] [42] [43], if only within given error limits.

Interestingly, the ascertained discrepancies appear to have their largest (joint) cause in the very short time-span (~10 yrs) that is traditionally assigned to Late Geometric Ia. Once introduced–apparently within ±10 yrs of the beginning of the Late Geometric–from that point in time backwards the dating offset remains effectively constant (in the order of 50–80 yrs) for several hundreds of years. At Sindos this ‘down-core’ propagation (wrongly younger) of a nominally constant error (cf. nearly parallel diagonal lines in Fig 11) is observable for all phases 7–11, with the notable exception of phase 6 (Late Geometric Ib). A memory-effect for a propagated dating error with this (actually quite small) magnitude would readily explain many of the observed discrepancies between the different chronologies, which are typically of given magnitude, and in particular the proposed updating of the Early Protogeometric at Assiros [4]. Unfortunately, it is not the initial occurrence of the dating error itself (in Late Geometric Ib), nor its first propagation steps (through the Middle Geometric), but rather more its down-core arrival in the Protogeometric that is difficult to judge. Namely, as can be taken from Fig 11, for ages older than ca.1000 calBC the GaussWM-derived dating errors from Sindos have values of ±50 yrs (95%) and higher. For all phases of the Protogeometric, this imprecision effectively hampers further meaningful comparisons.

## Conclusions

### Implications of the revision of Greek chronology on Aegean and Mediterranean archaeology

It is especially for the younger sections of the Aegean Early Iron Age, in particular for the Late Protogeometric and Geometric periods, that the new data from Sindos may have an impact in our general perception of the cultural and social transformations that took place in the Mediterranean. After a long period of time interregional contacts between Greece and the eastern Mediterranean began again from the Late Protogeometric onwards, while exchanges between the Aegean and Italy were restored in the following Geometric periods. From an archaeological perspective, these contacts are not only recognisable but also best-dated by means of pottery synchronisms. For example, circulation and consumption of drinking cups of the Middle Geometric and Late Geometric I periods overseas are in certain quarters perceived as indicators of Greek and Phoenician ‘pre- or early colonial’ activity. During those periods the Greeks, and probably also other people, are usually thought to have adopted the alphabet from the Phoenicians, appropriated new cultural and social habits such as the symposion, and prepared the ground for one of the most influential events in Mediterranean history, the ‘apoikismos’. According to the conventional chronology all this is thought to have taken place within a period of two generations, from 800 to 735 BC. Particularly significant in this respect is the allegedly short Late Geometric Ia phase, but which probably contains the bulk of the so-called ‘pre-colonial’ pottery in western Mediterranean. The proposed changes in the absolute Greek and consequently Mediterranean chronology may thus change our understanding of the timing and duration of (short) historical events, or (long) cultural processes that took place during the Early Iron Age. One of the rising questions is, for example, whether the transfer of writing really did take place within the time-span of only one generation, as is often assumed, and whether its adoption really did occur at some time in the 8th century calBC, or not already in the second half of the 9^th^ century calBC. Furthermore, the archaeologically visible restoration of contacts between the Aegean and the western Mediterranean, following the end of the Late Bronze Age, may not date to the first half of the 8th century calBC, but this occurred instead–based on the new evidence from Sindos–much earlier.

Any attempt to revise a well-established and widely respected–despite its deficiencies–chronological system is a major challenge. Dating revisions even on the seemingly small (multi-decadal) scale proposed here will naturally be perceived as inconvenient, in many respects, but herein especially due to their implications for the chronological systems and historical narratives of the Early Iron Age that are accepted as authoritative on a supra-regional scale. Instead, we put forward for the first time a series of radiocarbon dates for the Protogeometric and Geometric periods in the Aegean. The results contradict many of the interpretations that were based on, in our view, some largely ambiguous historiographic and archaeological dating methods. What is certainly needed are many further series of ^14^C-ages that derive from secure and well-published contexts in the Aegean and other regions, where Greek pottery was used, at best from long tell-stratigraphies and in combination with large-scale statistical pottery dating, based e.g. on Correspondence Analysis [34].
